# Cook Co. Hospital Clinic

**Published:** 1871-01

**Authors:** C. T. Fenn


Article III.—*Cook County Hospital Clinic.* Reported by C. T. Fenn, M.D.

Oct. 11. Pathological. Prof. Johnson presented a case of paralysis of the vaso-motor nerves of the left side—a feature rarely seen. An elderly man, he was taken suddenly, after a debauch, with partial paralysis of sensation and motion of the left side. There was no alteration in size. He gradually recovered both sensation and motion, but with the retention of other strange symptoms. Sensation is now slightly exalted; the left eye is inflamed; there is a sensible increase of temperature, and the whole left side is œdematous. Paralysis of the vaso-motor nerves of the sympathetic system is the only thing which could cause this change in appearance. The vessels transmit more blood; hence the increased heat, œdema, and finally hypertrophy.

Oct. 18. Surgical. Prof. Powell. 1. Case of injury, admitted the day before; trepanned soon after admission. He had fallen from a building, fifty feet, to the ground, striking on the right side of his head. He was comatose, and presented all the symptoms of compression. This is the third case of the kind within six weeks. The breathing, surface, pulse, and facial expression carefully observed. The only mark here is a superficial abrasion and a black eye. Coma and a black eye are, together, very grave symptoms. Nearly all the symptoms as laid down are sometimes absent. They are not so distinctly drawn between concussion and compression as the books show. Concussion recovers in a few hours; compression is more durable. There was dilatation of the pupil yesterday; there is a little to-day. He groans when his lame wrist is handled; pulse 82; respiration, not at all stertorous, is regular. He has spoken to-day. The danger is of inflammation. If he dies we shall find pus in the cavity of the arachnoid membrane. Use no perturbating treatment; no cathartic; no sedative. Treat symptoms as they occur; see to the urine; administer an enema, if necessary, every second day. Give a pint of beef tea every twenty-four hours. Trepanning is a grave operation; the injury is grave; but do not be deterred from using the trephine, especially if the injury is about the front of the head, and coma grows worse. Apply the trephine over bone of uniform thickness; don't wound the membrane.

2. Case of bursa on the wrist of a young girl. Ruptured by manipulation while an attendant was producing a tenotomy knife for subcutaneous puncture. Dressed with compress.

3. Case. Extensive circular sores at the knee. A woman aged thirty-nine, twice married, fell on the ice last winter, injuring her leg. The peculiarity of these sores is that they are circular, healing in one direction while they spread in another, and there are several. It is lupus exedens, and probably syphilitic. Best dressing—creosote ointment. Administer of iodide or bromide of potassium as much as she can bear.

4. Cases of venereal disease. These affections are not treated in the Hospital, but happening to get in from other causes, are reserved for exhibition. The three poisons of venereal disease are, 1, gonorrhœa; 2, chancroid, or local contagious ulcer; and 3, chancre. The first is local. The second is also local. The third is evidence that the system is infected. If you take any poison, as woorara or opium, even, and introduce within the skin, it is immediately felt. It is absolutely certain that the poison of syphilis is immediately absorbed. A chancre is proof of it. You will cauterize it to no purpose. Case before us. Let us make a diagnosis. Chancroid is a local disease. There may be an ulcer, it may be sloughing or persistent; there may be a bubo. The distinction between syphilis and chancroidal disease is very difficult. One of the principal points to be determined is the time from exposure to the development. Chancre has a certain period of incubation, two weeks. In chancroid there is no such period. It sometimes appears almost immediately, certainly within two or three days. Chancroid looks like a clear punch in the mucous membrane. If in the integument it is at first a pimple, then a follicle, then a papule, then a pustule, then a deep ulcer. The local ulcer is more terrible to look at, followed, nearly always, by suppurating glands in the groin. The local ulcer will inoculate; chancre will not. In chancre the enlargement of the gland is hard like a pebble beneath the skin; rarely suppurates. Be guarded, but if you see a suppurating bubo, you may be pretty sure it is the local ulcer. In chancre the patient is almost certain to have constitutional symptoms in from three weeks to six months. He will have all the symptoms of febrile action. If you examine the head you will find patches; the throat will be dusky and red. You may from this have developed any one of the skin diseases. There is most apt to be developed, in the throat and about the inner surface of the lips, mucous patches. He is apt to have falling of the hair, pain in the bones, and ever thereafter be subject to a repetition of all the forms. Don't give mercury till you know you have syphilis to treat; for, by giving it you may produce all the symptoms you wish to alleviate. The one produces all the symptoms of the other. Mercury is a dangerous remedy in some hands. It is dangerous to give it in persons run down by any disease. This is a case of syphilis, for the sores are not irregular, not large, only two or three of them. The groin has a solid gland, not large.

Case. To show the development of some constitutional symptoms certain forms of skin disease come later than others. Syphilitic rupia may appear very late, three years. It is an exceedingly destructive disease. This case looks like syphilitic rupia. He had a chancre eighteen days after exposure.

Case. Vegetations not venereal, result of a long prepuce. (Laughter, directed toward the side on which the ladies sat.) Direct the patient to be cleanly, and if he is not so, he is to be circumcised.

Case. Bony or sub-periosteal tumors following rupia. Give these cases preparations of iron and warm flannel. Do not give the alkalies, for they impoverish the blood.

Case. Syphilitic psoriasis; also, white patches. Treatment: Protiodide of Mercury, gr. iv; Iodide of Potassium, ʒ iv; Water, f ℥ viij. M. S. Give a teaspoonful three times daily. This will cure in six to eight weeks.

Case. Gonorrhœa, resulting in orchitis. Don't give injections, and don't be over anxious about checking these discharges, for you will be apt to get orchitis. It may lay the patient up. Treatment: Opium, to relieve pain. Open the bowels. Lotions to reduce the inflammation. In this case we will try penciling the surface of the scrotum with a solution of Nitrate of Silver, two scruples to the ounce, till the skin is vessicated. Our practice has been to strap these cases; but we will see how the new method works.

Case. Balanitic form of long prepuce getting well. He will have to be circumcised. These patients are all to be discharged. We cannot keep them here.

At the close of the hour the students were taken to the dead-house and instructed in the art of post mortem examination of the brain. Use the saw as you would trepan, being careful not to cut through the membrane. Familiarity with post mortem examinations is of use in giving one correct knowledge of the use of instruments. Perform as often as you can. Steps: 1. Part the hair over the top of the head from ear to ear. 2. Make an incision in the same line. 3. Invert both flaps of the scalp by pulling them from the scull. 4. Cut across the temporal muscles with a knife. 5. Start the saw all around the cranium. 6. Try with a chisel to see if it is through; you may break the inner table. You may use a hook. If the calvarium does not come off readily, know it is not quite through all around. Look at the inner surface of the calvarium; then the dura mater. Divide the dura mater with scissors on a line corresponding with the division of the skull—turn it back; examine the surface of the brain. There may be a post mortem congestion. Remove the brain. Replace the parts in the order of their removal. A strong suture should be taken through the divided temporal muscles, for the purpose of holding the skull-cap firmly in place. If the brain should not be replaced no deformity can be seen when the operation is complete.

Medical. Ross. Case. Typhoid fever. Diarhœa began in the middle of the second week. Shows great prostration by the position assumed in bed, and indisposition to move. No delirium; tongue glazed, dry and crackling. Characteristic spots present. A pulse that is permanently 110; is a bad case. We do not always get a frequent pulse in typhoid, but we invariably get increased temperature, as indicated by the thermometer. Give a pint of good beef tea, made from a pound of beef, and two pints of milk every twenty-four hours. Keep plenty of air, and the temperature of the room at 60°. Cover the patient with blankets. If the meteorism is great, besides turpentine emulsion, we may use stupes. If it should be very great, we may give turpentine by enema. Morphine to secure rest.

